# Age-related changes in human sperm DNA integrity

**DOI:** 10.18632/aging.102120

**Published:** 2019-08-13

**Authors:** Aleksandra Rosiak-Gill, Kamil Gill, Joanna Jakubik, Monika Fraczek, Lukasz Patorski, Dariusz Gaczarzewicz, Rafał Kurzawa, Maciej Kurpisz, Malgorzata Piasecka

**Affiliations:** 1 Department of Histology and Developmental Biology, Pomeranian Medical University in Szczecin, Szczecin 71-210, Poland; 2 Institute of Human Genetics, Polish Academy of Sciences, Poznan 60-479, Poland; 3 Department of Gynecology, Endocrinology and Gynecological Oncology, Pomeranian Medical University in Szczecin, Szczecin 71-252, Poland; 4 Department of Animal Reproduction, Biotechnology and Environmental Hygiene, West Pomeranian University of Technology, Szczecin 71-270, Poland; 5 Department of Procreative Health, Pomeranian Medical University in Szczecin, Szczecin 71-210, Poland; 6 VitroLive Fertility Clinic in Szczecin, Szczecin 70-483, Poland

**Keywords:** paternal age, standard semen characteristic, sperm DNA integrity, male fertility, TUNEL

## Abstract

Abnormal standard semen characteristics and reduced sperm chromatin maturity can appear with increasing male age. However, the influence of paternal age on semen parameters is still controversial. Therefore, this study was designed to estimate the influence of paternal age not only on conventional semen characteristics but also on sperm DNA integrity. This research was carried out on ejaculated sperm cells obtained from men (n = 1124) aged ≥40 y and <40 y. Our data revealed a decreased semen volume and an increased percentage of DFI (sperm DNA fragmentation index) in older men compared to younger men in the entire study cohort, in men with normozoospermia and in men with abnormal semen parameters. Moreover, there was a higher incidence of sperm DNA damage (>10% DFI, low fertility potential) in the groups of men aged ≥40 y than in the groups of men aged <40 y. Older men had over twice the odds ratio for high sperm DNA damage as younger men. Our findings suggest a detrimental effect of advanced paternal age on sperm chromatin integrity. The data show that the evaluation of sperm DNA has greater clinical utility than standard semen analysis in case of male fertility potential assessment.

## INTRODUCTION

Infertility has become a worldwide problem, affecting up to 20% of couples trying to conceive [1, 2]. In this context, a few important facts should be emphasized: 1) male factors (coexisting with female factors) contribute to infertility in up to 20–70% of cases, and one-third of these cases are due to male factors alone [1–3]; 2) an actual decline in semen quality over the past decades has been observed globally [4]; and 3) paternal age is rising, as an increasing number of men are decide to became a father at an older age [5, 6].

**Figure 1:**
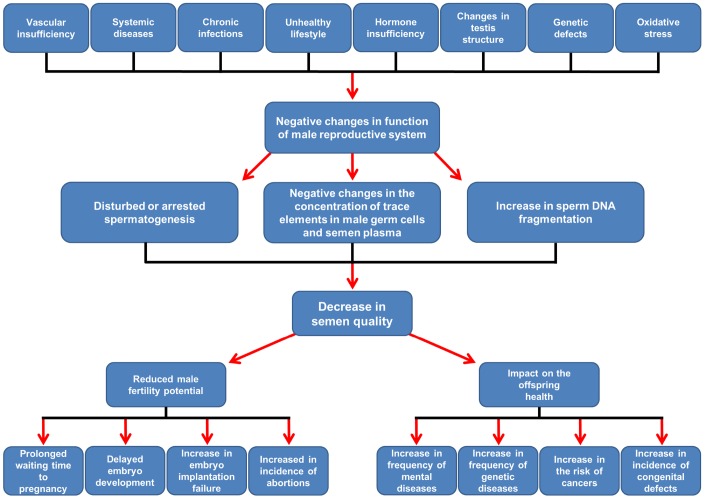
**Scheme of suggested age-related changes affecting the male reproductive system and their consequences for male fertility and offspring health. Details in the text.** According to Rosiak et al. [6, 9], modified.

It is known that the risk of poor reproductive outcomes can increase with a male age of >40 or even >35 y, commonly classified as advanced age. Age-dependent changes in male organism (*e.g.* vascular sufficiency, increase in incidence of systemic diseases and infections, disorders of histological structure of testes, decreased levels of sex hormones, oxidative stress, *de novo* mutations) are deleterious and the consequences of advanced paternal age include a prolonged waiting time to pregnancy, delayed embryo development in *in vitro* conditions, an increased incidence of embryo implantation failure and abortions, pregnancy problems and live birth outcome [7–9] (Figure 1). In addition, advanced paternal age also seems to affect children's health. There is a positive correlation between paternal age and the incidence of mental deprivation of offspring, such as those associated with the autism spectrum and diseases such as schizophrenia, especially when the paternal age is ≥40–50 y [10–13]. Furthermore, the frequency of genetic disorders, such as Klinefelter syndrome [14]; Down syndrome, when mother age is >35 y [15]; and autosomal dominant diseases such as Marfan syndrome (men >40 y), Pfeiffer and Crouzon syndrome (men >50 y), Apert syndrome (men >37 y), achondroplasia and neurofibromatosis type 1 rises in children of fathers >40 y. Unfortunately, the risk of central nervous system and breast cancers as well as leukaemia is growing [12]. More often, children with heart defects (*e.g.*, ventricular septal defects, atrial septal defects, large vessel transposition), neural tube defects, anencephaly and tracheo-oesophageal fistula have been born to men >35, 40, and 45 y of age [13] (Figure 1).

Age-related negative changes in sperm quality are observed in men over 35 y of age, and with age (>40 y), these changes become more pronounced [6]. Deterioration of semen quality is visible in the ejaculate volume, sperm count, motility, vitality and sperm morphology [16, 17]. It should be highlighted that the detrimental effect of ageing is also noted in the sperm chromatin status [18, 19]. However, negative changes in all semen parameters are not always observed at the same time. Therefore, it is difficult to definitively determine the influence of age on male gonad function and semen quality. Due to unclear, ambiguous and controversial data, our study was designed to estimate the impact of paternal age not only on conventional semen characteristics but also on sperm nuclear DNA integrity.

## RESULTS

According to other authors [20–24] from the entire cohort of men and in the groups of men with normozoospermia and abnormal semen parameters, we decided to designate two subgroups of men: ≥40 y and <40 y of age. The obtained data revealed the differences in selected sperm conventional characteristics and sperm DNA fragmentation between age groups. The older men had a significantly lower ejaculate volume, percentage of sperm cells with normal morphology, and percentage of male gametes with non-progressive motility and a higher percentage of DFI (sperm DNA fragmentation index) than the younger men (Table 1). In turn, in the older subgroup with normozoospermia, the ejaculate volume and TZI (teratozoospermia index) were significantly decreased, but the percentage of spermatozoa with progressive motility, percentage of eosin-negative spermatozoa and percentage of male gametes with DNA strand breaks were significantly increased compared to those of the younger subgroup with normozoospermia (Table 2). Moreover, the older subgroup with abnormal semen parameters had a significantly lower ejaculate volume and percentage of sperm cells with non-progressive motility and a higher percentage of DFI than the younger subgroup with abnormal semen characteristics (Table 3).

**Table 1:** Descriptive statistics and comparisons of standard semen parameters and DFI between men aged <40 y and ≥40 y.

**Parameters n median (range) mean±SD**	**< 40 y (n = 902)**	**≥ 40 y (n = 222)**	**p**
**Age (y)**	902 32.00 (18.00–39.00) 31.05 (5.07)	222 43 (40.00–70.00) 45.41 (6.11)	0.00001
**Semen volume (mL)**	902 3.35 (0.50–12.00) 3.54 (1.66)	222 3.00 (0.15–10.00) 3.10 (1.63)	0.00012
**Sperm concentration (×10^6^/mL)**	902 33.56 (0.001–347.700) 47.03 (44.46)	222 39.50 (0.10–216.00) 50.97 (46.57)	0.40039
**Total number of spermatozooa (x10^6^)**	902 107.70 (0.002–2109.75) 166.22 (191.67)	222 105.50 (0.15–931.50) 148.72 (154.44)	0.2374
**Morphologically normal spermatozoa (%)**	902 4.00 (0.00–34.00) 5.63 (5.80)	222 4.00 (0.00–28.00) 4.36 (4.14)	0.03200
**TZI**	845 1.63 (1.02–2.62) 1.65 (0.25)	218 1.61 (1.21–2.33) 1.62 (0.21)	0.11902
**Progressive motility (%)**	902 40.00 (0.00–90.00) 39.32 (19.73)	222 41.00 (0.00–88.00) 40.62 (21.76)	0.40505
**Non-progressive motility (%)**	902 14.00 (0.00–59.00) 16.38 (9.80)	222 11.00 (0.00–54.00) 13.71 (8.86)	0.00013
**Immotile sperms (%)**	902 41.00 (2.00–100.00) 44.27 (19.15)	222 44.00 (1.00–99.00) 45.66 (23.12)	0.61195
**Eosin-negative spermatozoa – live cells (%)**	897 68.00 (0.00–98.00) 65.18 (17.73)	220 66.00 (2.00–99.00) 64.41 (20.76)	0.95651
**Hos-test-positive spermatozoa – live cells (%)**	631 62.00 (0.00–93.00) 59.25 (17.95)	190 65.00 (10.00–94.00) 60.24 (18.19)	0.47225
**Round cells concentration (x 10^6^/mL)**	862 0.16 (0.00–37.34) 0.74 (2.16)	216 0.10 (0.00–11.00) 0.51 (1.22)	0.21214
**Peroxidase-positive cells (x10^6^/mL)**	869 0.00 (0.00–11.70) 0.37 (1.06)	216 0.00 (0.00–6.00) 0.28 (0.74)	0.27913
**DFI (%)**	248 10.82 (0.77–65.14) 13.37 (10.31)	88 18.47 (0.76–75.94) 19.77 (13.12)	0.00001

DFI – sperm DNA fragmentation index, n – number of subjects, SD – standard deviation, TZI – teratozoospermia index, p – significance of differences between compared groups, Mann–Whitney U test

**Table 2:** Descriptive statistics and comparisons of standard semen parameters and DFI between men aged <40 y and ≥40 y with normozoospermia.

**Parameters n median (range) mean±SD**	**< 40 y (n = 367)**	**≥ 40 y (n = 81)**	**p**
**Age (y)**	367 31.00 (18.00–39.00) 30.06 (5.25)	81 43.00 (40.00–53.00) 43.97 (4.10)	0.00001
**Semen volume (mL)**	367 3.50 (0.80–12.00) 3.86 (1.77)	81 2.80 (0.80–8.70) 3.30 (1.68)	0.00219
**Sperm concentration (×10^6^/mL)**	367 56.00 (8.90–347.70) 68.05 (50.58)	81 59.00 (9.20–216.00) 72.49 (46.33)	0.24677
**Total number of spermatozooa (x10^6^)**	367 187.00 (39.00–2109.75) 251.49 (235.54)	81 171.00 (39.00–931.50) 225.14 (170.72)	0.56813
**Morphologically normal spermatozoa (%)**	367 7.00 (4.00–34.00) 9.40 (6.33)	81 7.00 (4.00–28.00) 7.62 (4.31)	0.10451
**TZI**	350 1.54 (1.08–2.49) 1.54 (0.20)	79 1.49 (1.21–1.89) 1.49 (0.15)	0.03818
**Progressive motility (%)**	367 51.00 (32.00–90.00) 52.41 (12.94)	81 55.00 (32.00–88.00) 56.22 (13.67)	0.02615
**Non-progressive motility (%)**	367 14.00 (1.00–44.00) 15.27 (8.03)	81 11.00 (3.00–33.00) 13.58 (7.22)	0.11685
**Immotile sperms (%)**	367 32.00 (2.00–66.00) 32.44 (11.63)	81 30.00 (1.00–58.00) 30.19 (13.05)	0.23924
**Eosin-negative spermatozoa – live cells (%)**	366 74.00 (35.00–98.00) 73.19 (11.34)	81 80.00 (46.00–99.00) 76.37 (13.23)	0.01771
**Hos-test-positive spermatozoa – live cells (%)**	252 69.50 (28.00–92.00) 67.40 (11.91)	73 69.00 (40.00–94.00) 69.49 (11.87)	0.22170
**Round cells concentration** **(x 10^6^/mL)**	351 0.15 (0.00–37.34) 0.83 (2.88)	79 0.10 (0.00–11.70) 0.59 (1.52)	0.97768
**Peroxidase-positive cells (x10^6^/mL)**	354 0.00 (0.00–11.56) 0.28 (0.86)	79 0.00 (0.00–6.00) 0.25 (0.75)	0.81045
**DFI (%)**	116 11.45 (0.77–65.14) 12.74 (9.48)	44 14.77 (1.00–40.52) 17.68 (10.45)	0.00518

DFI – sperm DNA fragmentation index, n – number of subjects, SD – standard deviation, TZI – teratozoospermia index, p – significance of differences between compared groups, Mann–Whitney U test

**Table 3:** Descriptive statistics and comparisons of standard semen parameters and DFI between men aged <40 y and ≥40 y with abnormal semen parameters.

**Parameters n median (range) mean±SD**	**< 40 y** **(n = 535)**	**≥ 40 y** **(n = 141)**	**p**
**Age (y)**	535 32.00 (18.00–39.00) 31.72 (4.83)	141 44.00 (40.00–70.00) 46.24 (6.88)	0.00001
**Semen volume (mL)**	535 3.00 (0.50–10.00) 3.32 (1.55)	141 3.00 (0.15–10.00) 2.99 (1.60)	0.01952
**Sperm concentration (×10^6^/mL)**	535 22.50 (0.001–238.30) 32.62 (32.71)	141 22.30 (0.10–210.00) 38.61 (42.14)	0.55474
**Total number of spermatozooa (x10^6^)**	535 62.50 (0.002–800.00) 107.74 (124.64)	141 59.50 (0.15–689.00) 104.82 (125.21)	0.44229
**Morphologically normal spermatozoa (%)**	535 2.00 (0.00–22.00) 3.04 (3.58)	141 2.00 (0.00–11.00) 2.48 (2.61)	0.24004
**TZI**	495 1.72 (1.02–2.62) 1.73 (0.24)	139 1.68 (1.26–2.33) 1.70 (0.21)	0.16066
**Progressive motility (%)**	535 27.00 (0.00–84.00) 30.34 (18.53)	141 30.00 (0.00–88.00) 31.65 (20.47)	0.59229
**Non-progressive motility (%)**	535 15.00 (0.00–59.00) 17.14 (10.79)	141 11.00 (0.00–54.00) 13.79 (9.70)	0.00033
**Immotile sperms (%)**	535 50.00 (6.00–100.00) 52.38 (19.09)	141 52.00 (4.00–99.00) 54.55 (22.99)	0.21761
**Eosin-negative spermatozoa – live cells (%)**	531 64.00 (0.00–98.00) 59.66 (19.19)	139 58.00 (2.00–97.00) 57.44 (21.21)	0.23521
**Hos-test-positive spermatozoa – live cells (%)**	379 57.00 (0.00–90.00) 53.84 (19.21)	117 57.00 (11.00–90.00) 54.47 (19.09)	0.84956
**Round cells concentration** **(x 10^6^/mL)**	511 0.20 (0.00–14.68) 0.68 (1.48)	137 0.10 (0.00–7.03) 0.46 (1.00)	0.10633
**Peroxidase-positive cells (x10^6^/mL)**	515 0.00 (0.00–11.70) 0.43 (1.17)	137 0.00 (0.00–4.80) 0.30 (0.74)	0.21906
**DFI (%)**	132 10.54 (1.00–60.20) 13.92 (10.99)	44 20.45 (0.76–75.94) 21.85 (15.18)	0.00120

DFI – sperm DNA fragmentation index, n – number of subjects, SD – standard deviation, TZI – teratozoospermia index, p – significance of differences between compared groups, Mann–Whitney U test

Furthermore, based on reports by other authors [25–27], we decided to designate a cut-off point for DNA damage of 10%. This threshold is considered to indicate high sperm DNA damage and is referred to as low fertility potential. The older men in the entire cohort, the group of men with normozoospermia and the group of men with abnormal semen parameters had a significantly higher prevalence of sperm cells with DNA damage >10% than the younger men in these groups (subjects: 72.73% vs. 53.63%, 75.00% vs. 55.17%, 70.46% vs. 52.27%, respectively) (Tables 4–6). Likewise, men ≥40 y of age had an OR (odds ratio) for having a high level of sperm DNA damage that was over 2-fold higher than that of the men <40 y in both the entire cohort and the group of men with normal and abnormal standard semen parameters (OR: 2.3058, OR: 2.4375, OR: 2.1773, respectively) (Tables 7–9).

**Table 4:** Prevalence of DFI in the group of men aged ≥40 y and <40 y in the entire cohort.

**Group**	**DFI (%)**	
	**0–10% n(%)**	**>10% n(%)**
**<40 y (n = 248)**	115(46.37)	133(53.63)
**≥40 y (n = 88)**	24(27.27)*	64(72.73)*

DFI – sperm DNA fragmentation index, n – number of subjects. *Significant difference between compared groups at p = 0.0018; chi-squared test

**Table 5:** Prevalence of DFI in the group of men aged ≥40 y and <40 y with normal standard semen parameters.

**Group**	**DFI (%)**	
	**0–10% n(%)**	**>10% n(%)**
**<40 y (n = 116)**	52(44.83)	64(55.17)
**≥40 y (n = 44)**	11(25.00)*	33(75.00)*

DFI – sperm DNA fragmentation index, n – number of subjects. *Significant difference between compared groups at p = 0.0223; chi-squared test

**Table 6:** Prevalence of DFI in the group of men aged ≥40 y and <40 y with abnormal standard semen parameters.

**Group**	**DFI (%)**	
	**0–10% n(%)**	**>10% n(%)**
**<40 y (n = 132)**	63(47.73)	69(52.27)
**≥40 y (n = 44)**	13(29.54)*	31(70.46)*

DFI – sperm DNA fragmentation index, n – number of subjects. *Significant difference between compared groups at p = 0.0355; chi-squared test.

**Table 7:** Odds ratio (OR) for DFI in the group of men aged ≥40 y compared to men <40 y in the entire cohort.

	**< 40 y (n = 248)** **n(%)**	**≥ 40 y (n = 88)** **n(%)**	**OR (95%CI)**
**DFI 0–10%**	115(46.37)	24(27.27)	0.4337* (0.2549 to 0.7378)
DFI >10%	133(53.63)	64(72.73)	2.3058* (1.3553–3.9228)

DFI – sperm DNA fragmentation index, n – number of subjects. *Statistical significance at p = 0.0021; 95% CI – 95% confidential interval

**Table 8:** Odds ratio (OR) for DFI in the group of men aged ≥ 40 y compared to men <40 y with normal standard semen parameters.

	**<40 (n = 116)** **n(%)**	**≥40 y (n = 44)** **(n%)**	**OR (95%CI)**
**DFI 0–10%**	52(44.83)	11(25.00)	0.4103* (0.1891–0.8899)
**DFI >10%**	64(55.17)	33(75.00)	2.4375* (1.1237–5.2871)

DFI – sperm DNA fragmentation index, n – number of subjects.*statistical significance at p = 0.0241; 95% CI – 95% confidential interval

**Table 9:** Odds ratio (OR) for DFI in the group of men aged ≥40 y compared to men <40 y with abnormal standard semen parameters.

	**<40 y (n = 132)** **n(%)**	**≥40 y (n = 44)** **n(%)**	**OR (95%CI)**
**DFI 0–10%**	63(47.73)	13(29.54)	0.4593* (0.2209–0.9551)
DFI >10%	69(52.27)	31(70.46)	2.1773* (1.0470–4.5278)

DFI – sperm DNA fragmentation index, n – number of subjects. *statistical significance at p = 0.0373; 95% CI – 95% confidential interval

## DISCUSSION

### Age-dependent decrease in selected semen characteristics

In our study, we wanted to verify the influence of ageing on semen quality. Therefore, based on the suggestions of other authors [20, 22, 23] men ≥40 y and <40 y of age were distinguished in the entire cohort of subjects, in the group of men with normozoospermia and in the group of men with abnormal semen parameters. We observed a significant decrease in semen volume with ageing. This parameter declined after ≥40 y of age in all study groups. The obtained results were in line with those of other researchers [16, 28]. However, an association between age and semen volume was not always found [7, 20, 23].

In accordance with most of the published reports [7, 16], we found that ageing is characterized by a statistically significant decrease in the percentages of morphologically normal spermatozoa (in the entire cohort). Stone et al. [16] demonstrated a decline in the percentage of sperm cells with normal morphology in men aged >40 y. Similar results were obtained by other authors [7, 29, 30] who have shown that the percentage of sperm cells with normal structure decreased significantly in men aged >50–79 y. However, Park et al. [31] and Kaarouch et al. [20] did not show a relationship between sperm morphology and paternal age.

In the present study, no significant age-related decreases in the sperm concentration, total sperm count, sperm motility or vitality were revealed, which was consistent with the data obtained by some researchers [7, 20, 28, 31]. However, some authors have found these parameters to be dependent on age [16, 17]. What is interesting and surprising in group of men with normozoospermia, older men had slightly but significantly higher percentage of sperm progressive motility and vitality and those results highlighted how complicate could be estimation of age influence on basic semen parameters.

### Age-dependent increase in sperm DNA fragmentation

To undertake more sophisticated evaluations of associations between male ageing and sperm quality, we performed DNA fragmentation as measured by the TUNEL (terminal deoxynucleotidyl transferase-mediated dUTP nick end labelling)/PI (propidium iodide) staining. In contrast to the comparison of standard semen parameters, in all groups, older men (≥40) had higher percentage of DFI that younger men. Several studies have presented similar data [18, 20, 23, 32]. The impact of age on sperm DNA integrity was revealed by Kaarouch et al. [20] and Alshahrani et al. [23], who showed that men ≥40 y had a significantly higher percentage of DFI than younger men. Similarly, Vagnini et al. [32] observed significant differences in the percentage of sperm with fragmented sperm DNA between patients ≤35 y vs. 36–39 y and vs. ≥40 y. Additionally, Plastira et al. [18] compared the sperm DNA integrity of men with oligoasthenoteratozoospermia and men with normozoospermia in two age-dependent groups: 24–34 y and 35–45 y. The researchers showed a significantly higher percentage of DFI in the group of older men with oligoasthenoteratozoospermia than the group of younger men. Moreover, they found significant correlations between age and the percentage of sperm cells with damaged chromatin. However, the authors did not find these differences or correlations in the group of men with normozoospermia. Similar results were obtained by Winkle et al. [19]; the group of men aged ≥40 y with abnormal standard semen parameters had a significantly higher percentage of DFI than subjects aged 36–39 y.

Based on Hallak [25], Borini et al. [26] and Benchaib et al. [27], we assumed that a >10% DFI is related to declining male fertility potential. The incidence of men with >10% DFI was higher in the group of subjects aged ≥40 y regardless of conventional characteristics of sperm. Moreover, older men had over double the risk of having >10% DFI compared to younger men.

### Possible pathological mechanism of age-related changes in semen quality

The pathological mechanism responsible for age-dependent patterns of decline in quantitative and qualitative semen parameters has not been fully elucidated and seems to be multifactorial [8, 33, 34]. However, it is known that a decrease in sperm quality may result from age-related excessive generation of ROS (reactive oxygen species), sperm-limited antioxidant defences and the stimulation of sperm damage by oxidative stress. It is well documented that sperm nuclear DNA fragmentation is positively correlated with the overproduction of ROS [17, 35, 36]. Additionally, male ageing is often associated with defective sperm DNA remodelling mechanisms that result in poorly packaged chromatin and a decreased ability to repair DNA strand breaks. It is therefore understandable why older males are more susceptible to oxidative attack and more prone to errors during spermiogenesis, leading to a natural decline in male fertility [35–39] (Figure 1).

### Limitations of the study

Some limitations of our study should be addressed. Firstly, most of our subjects were in reproductive age, under 40 years and in all comparisons disproportion of groups size existed. In statistical analysis it is significant, and changes in groups proportions could influence the results. Secondly, we would like highlighted the fact that VitroLive Fertility Clinic and the Andrology Laboratory of Department of Histology and Developmental Biology are localized in the same city. Almost all of participants belong to the same nation and live in a similar environment. Because presented paper concern a global problem it would be valuable to performed multi-central research involving clinics and laboratories from other geographic regions. Thirdly, single assay was used to analyse the sperm DNA damage. DFI was verified only by TUNEL method, however this assay has strong clinical utility [25–27]. Further research with more DNA tests seems to be justified.

### Final remarks

Our findings suggest a significant detrimental effect of advanced paternal age on sperm chromatin integrity because they reveal a significantly higher incidence of men with >10% DFI in the group of subjects aged ≥40 y. Furthermore, regardless of a standard semen analysis, groups of men after age 40 y are more than twice as likely to have >10% DFI than the groups of younger men and are recognized as having low fertility potential [25–27]. Therefore, our findings are consistent with the results of other authors reporting that a paternal age >40 y, commonly classified as advanced age, can be associated with a higher risk of reproductive failure [18-20, 23, 32]. It should be highlighted that the age-related changes are visible not only in selected conventional semen characteristics but also in the sperm chromatin status. It is particularly important because many authors have shown that the assessment of nuclear sperm DNA quality has greater clinical utility than standard semen analysis and better discriminates men with normal fertility potential from men with reduced fertility potential.

## METHODS

### Study population

To perform this study, 1124 men (median: 33 y of age) were enrolled. The patients were partners in an infertile couples who were treated in the VitroLive Fertility Clinic (Szczecin, Poland) (n = 763) while volunteers with unknown fertility status (n = 361) reported to the Andrology Laboratory of Department of Histology and Developmental Biology (Pomeranian Medical University in Szczecin, Poland) for assessment of basic semen parameters. For all participants, the exclusion criteria included the following: azoospermia; a history of testicular torsion, cryptorchidism, testicular injury or cancer; varicocele; co-existing systemic disease; and a history of mumps. The ethics committee of the Pomeranian Medical University, Szczecin, Poland, approved this study protocol (ethical authorization number: KB-0012/10/14).

### Standard semen analyses

The semen samples were obtained by masturbation after 2–7 days of sexual abstinence. The conventional semen parameters were assessed according to the World Health Organization 5^th^ edition criteria [40] and performed in the Andrology Laboratory. Sperm motility (total, progressive and non-progressive motility) and vitality (eosin staining and hypoosmotic swelling test–HOS test) were determined under a contrast phase microscope and in bright field of light microscope, respectively (Primo Star, Zeiss, Germany). The sperm concentration was assessed with the improved Neubauer haemocytometer (Heinz Hernez Medizinalbedarf GmbH, Hamburg, Germany). The sperm morphology (including the TZI) was evaluated using the Papanicolaou staining method under a bright light microscope (CX 31 Olympus Optical Co., Ltd., Tokyo, Japan). Normozoospermia was considered according to the following criteria: sperm concentration ≥15 mln/mL, total number of sperm ≥39 mln, sperm progressive motility ≥32% and morphology ≥4%. Moreover, the TZI, vitality (eosin-negative and HOS-positive sperm cells) and concentration of round and peroxidase-positive cells (leukocytes) were evaluated. The standard semen evaluation was performed using a bright light microscope (CX31 Olympus Optical Co., Ltd., Tokyo, Japan). In the entire studied population the following seminological abnormalities of the standard sperm parameters were distinguished: normozoospermia (n = 448), asthenozoospermia (abnormal sperm motility, n = 107); asthenoteratozoospermia (abnormal sperm motility and morphology, n = 127); oligozoospermia (abnormal sperm number, n = 40); oligoasthenozoospermia (abnormal sperm number and motility, n = 31); oligoasthenoteratozoospermia (abnormal sperm number, motility and morphology, n = 133); oligoteratozoospermia (abnormal sperm number and morphology, n = 45) and teratozoospermia (abnormal sperm morphology, n = 193).

### Evaluation of sperm DNA fragmentation index (DFI)

Nuclear DNA strand breaks in sperm cells were identified by TUNEL assay (terminal deoxynucleotidyl transferase-mediated dUTP nick end labelling)/PI (propidium iodide) assay using the FlowTACS^TM^ Apoptosis Detection Kit (Trevigen, Inc., Gaithersburg, MD, USA) according to the manufacturer’s instructions. The liquefied ejaculate was centrifuged for 15 min at 300 g, and the pellet was washed twice in PBS (phosphate-buffered saline, Sigma Aldrich GmbH, St. Louis, USA) and fixed in 1% (v/v) formalin for 15 min at 4°C. After fixation, the pellet was washed twice (5 min, 300 g), resuspended with ice-cold 75% (v/v) ethanol and stored at -20°C for no less than two months. On the day of the test, samples were washed twice in PBS to remove the ethanol. Then, spermatozoa were permeabilized with Cytonin for 15 min at room temperature. After washing, the labelling reaction was performed.

The TUNEL test was based on the incorporation of substrate, biotin-labelled deoxynucleotide triphosphate (biotin-dNTP), into the free 3’-OH residues of the DNA fragments in the presence of terminal deoxynucleotidyl transferase (TdT). DNA breakage in the insertion sites of biotin-dNTP was identified by means of fluorescein isothiocyanate (FITC)-labelled streptavidin with a strong affinity for biotin. A negative control was obtained by omitting TdT from the reaction mixture, and a positive control was obtained by incubating sperm cells with DNase I. The TUNEL-positive spermatozoa (FITC-streptavidin-biotin-dNTP-labelled cells) were checked with a fluorescence microscope (BX41 Olympus Optical Co., Tokyo, Japan). After the TUNEL labelling reaction, previously permeabilized sperm cells (0.1% Triton X-100 in 0.1% sodium citrate) were stained with propidium iodide (PI) to discriminate apoptotic cells from necrotic cells in the flow cytometry analysis.

### Flow cytometry measurements and data analysis

The verification of the incidence of sperm cells with SDF was performed using a Beckman Coulter flow cytometer (Cell LabQuanta SC MPL, Beckman Coulter, Fullerton, CA, USA) equipped with a 488 nm argon-ion laser. For each analysis, at least 10,000 events were collected at a flow rate of 150–250 events/s. The analysis of the results was performed using Cell LabQuanta SC MPL Analysis software (Beckman Coulter). The sperm population was gated on the basis of measurements of electronic volume (EV, parameter depends on cell size) and side scatter (SS, parameter depends on cellular granules). The green (480–550) and red (590–670) fluorescence signals were measured simultaneously using the FL1 and FL3 channels, respectively. The fluorescent data were obtained at a fixed gain setting in logarithmic (FL1, FL3) mode.

**Figure 2:**
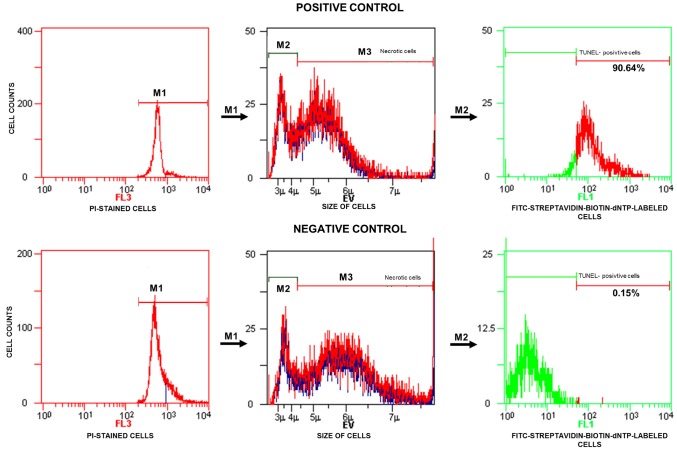
**Representative histograms obtained for the positive (with DNase I) and negative (without TdT) controls and strategy applied in the TUNEL/PI flow cytometry analysis.** In the fluorescence histograms, markers (M1, M2, M3) are set to exclude necrotic sperm cells and calculate the percentage of TUNEL-positive sperm cells (with fragmented DNA) read from the M2 marker. FL3 – red fluorescence channel for PI-stained cells, FL1 – green fluorescence channel for FITC-streptavidin-biotin-dNTP-labelled cells, TdT – terminal deoxynucleotidyl transferase, PI – propidium iodide, TdT – terminal deoxynucleotidyl transferase, TUNEL – terminal deoxynucleotidyl transferase-mediated dUTP nick end labelling.

**Figure 3:**
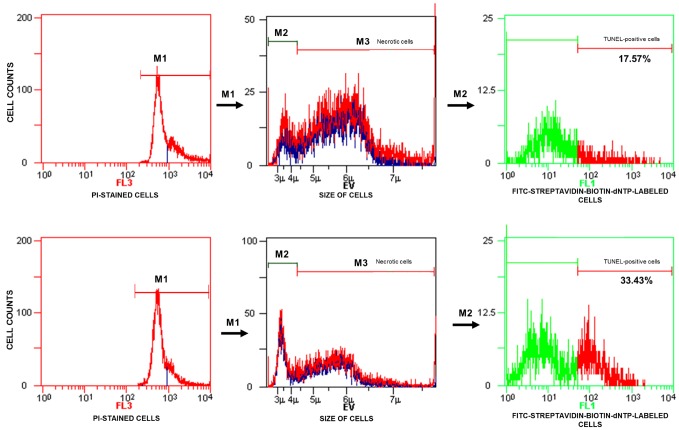
**Representative histograms obtained for test samples.** The calculation of TUNEL-positive sperm cells (with fragmented DNA) based on data analysis presented in Figure 1. FL3 – red fluorescence channel for PI-stained cells, FL3 – red fluorescence channel for PI-stained cells, FL1 – green fluorescence channel for FITC-streptavidin-biotin-dNTP-labelled cells, TUNEL – terminal deoxynucleotidyl transferase-mediated dUTP nick end labelling, PI – propidium iodide.

TUNEL/PI data analysis was performed according to the manufacturer’s instructions. The analyser threshold in the EV channel was adjusted to exclude debris and sperm aggregates from the flow cytometry analysis. The sperm-specific events were positively gated on the EV/SS dot plot. The calculation of TUNEL-positive spermatozoa with fragmented nuclear DNA (emitting green fluorescence) was based on positive (with DNase I) and negative (without TdT) histograms obtained for PI-stained spermatozoa (emitting red fluorescence). The staining of sperm cells with PI was performed after their permeabilization, and the positive and negative controls were generated for flow cytometry analysis to 1) exclude necrotic cells with fragmented DNA and 2) set a threshold to separate apoptotic sperm cells with DNA damage from cells with normal DNA integrity (Figure 2). The threshold was translated into histograms of test samples. The total events exhibiting green fluorescent intensities higher than the set threshold were considered apoptotic TUNEL-positive spermatozoa and expressed as a percentage, while the events emitting green fluorescence below the threshold were considered TUNEL-negative sperm cells displaying only background fluorescence (Figure 3).

### Statistical analysis

Statistical analysis was carried out using the software Statistica version 13.3 (StatSoft, Poland) and MedCalc version 15.2.2 (MedCalc Software, Belgium). Two independent group comparisons were performed using the Mann–Whitney U test for continuous variables. A chi-square test was used to compare the categorical data. The quantitative variables are expressed as the median (range) and means ± standard deviations (SDs), while qualitative data are presented as percentages. To define a risk for having high sperm DNA damage, an OR was calculated and is presented with the 95% confidence interval (CI) and p value. For all statistical tests, a p value of 0.05 was deemed significant.
